# Two decades of vagus nerve stimulation for stroke: a bibliometric analysis

**DOI:** 10.3389/fneur.2025.1531127

**Published:** 2025-04-04

**Authors:** Jiao Deng, Zhen Yang, Qing Mei Wang, Zhi Gang Lv

**Affiliations:** ^1^Department of Rehabilitation Medicine, Changzhou Hospital of Traditional Chinese Medicine, Changzhou, China; ^2^Physical Activity, Sports & Health Research Group, Faculty of Movement and Rehabilitation Sciences, KU Leuven, Leuven, Belgium; ^3^Stroke Biological Recovery Laboratory, Department of Physical Medicine and Rehabilitation, Spaulding Rehabilitation Hospital, The Teaching Affiliate of Harvard Medical School, Boston, MA, United States

**Keywords:** VNS, stroke, rehabilitation, knowledge map, bibliometric analysis

## Abstract

**Background:**

Stroke is a major global health concern, imposing significant medical and social burdens. Vagus nerve stimulation (VNS), an emerging neuromodulation technology, has shown potential in the treatment of stroke. This bibliometric analysis aims to explore the knowledge structure and research trends in the field of VNS for stroke from 2004 to 2024.

**Methods:**

Publications were retrieved from the Web of Science Core Collection. CiteSpace and VOSviewer were used to conduct bibliometric analyses, including author productivity, institutional contributions, and emerging research themes etc.

**Results:**

A total of 191 eligible publications were analysed. Kilgard, M. P., and Hays, S. A. were the most prolific authors, each contributing 26 publications. The USA (96 publications), China (69 publications), and Scotland (17 publications) were the most prolific countries. The University of Texas at Dallas (33 publications) was the most prolific institution, followed by Chongqing Medical University (19 publications) and the University of Glasgow (15 publications). Future research is expected to focus on: (1) neurophysiological mechanisms of VNS in stroke recovery; (2) synergistic effects of VNS with other rehabilitation therapies; (3) comparative efficacy of non-invasive transauricular VNS versus invasive VNS; (4) safety and effectiveness of VNS for post-stroke functional impairments beyond motor rehabilitation; and (5) optimisation of VNS parameters for stroke treatment.

**Conclusion:**

The field of VNS for stroke has experienced steady growth over the past two decades. This bibliometric analysis provides valuable insights to guide future research, clinical applications, and policy developments.

## 1 Introduction

Globally, stroke has been recognized as the second leading cause of death and the third leading cause of disability (1). Evidence from the Global Burden of Disease study shows that the number of patients with strokes increased by 70% from 1990 to 2019 (2), with a rising incidence among young and middle-aged people under 55-years-old. Moreover, The Lancet Neurology Commission estimate that global stroke-related deaths will increase by 50% from 2020 to 2050, with the number of people living with disabilities expected to rise by about 30% (3). As the aging population increasing, the burden of stroke will escalate significantly, presenting serious challenges to governments and healthcare globally. However, despite continuous advancements in rehabilitation treatments, effectively restoring patients’ functional abilities remains a major issue in stroke management.

Recently, vagus nerve stimulation (VNS), as an innovative neuromodulation technology, has shown potential in stroke rehabilitation by stimulating the vagus nerve to regulate central nervous system function. In 2021, the US Food and Drug Administration (FDA) approved the application of VNS combined with rehabilitation exercises for the treatment of chronic stroke (4). VNS can be administered through either invasive or non-invasive approaches. Invasive VNS (iVNS) involves costly surgery to implant electrodes in the left cervical vagus nerve, which are then stimulated by a pulse generator. Non-invasive VNS, also known as transauricular VNS (tVNS), typically includes transcutaneous VNS via the ear (taVNS) or cervical VNS (tcVNS) (5). Ventureyra (6) first identified tVNS as a novel non-invasive brain modulation technology. tVNS involves the using low-frequency pulse currents to stimulate the auricular branch of the vagus nerve. Moreover, safety, cost-effectiveness, easy to administer, and high acceptability are the advantages of tVNS (7). Recently, a recent randomized controlled trial found that the relative growth of ischemic lesions on 24-h diffusion-weighted magnetic resonance imaging (MRI) was 63% in the tVNS group, while it was 184% in the sham stimulation group (*p* = 0.109). This suggests that tVNS has a neuroprotective effect in acute stroke (8). Moreover, other studies have shown that taVNS combined with motor rehabilitation can enhance motor function (9), proprioception, and light touch sensation (10) in chronic stroke patients.

Bibliometric analysis holds a significant place in medical research, primarily due to its ability in generating broad evidence synthesis in a specific research field. In the research evolution framework, studies can be categorized into two types: original research and evidence synthesis. Evidence synthesis can be further divided into two forms: deep synthesis (i.e., systematic reviews and meta-analyses) (11) and broad synthesis (i.e., bibliometric analysis) (12). Deep synthesis is typically used to address specific research questions, for example, focusing on the effectiveness of a particular intervention on certain health-related outcomes, thus providing a precise conclusion. In contrast, broad synthesis is suited for analysing the overall knowledge structure of a research field and predicting future research trends.

In the field of stroke, while there have been numerous deep syntheses on VNS technology, there is a lack of broad synthesis as bibliometric analyses. Therefore, it is necessary to conduct a comprehensive bibliometric analysis in the field of VNS for stroke. The current study aims to provide a deep understanding of current research hotspots, trends, and the overall knowledge structure. This work will not only identify key research directions and potential gaps but also serve as a valuable reference for future research collaborations (13).

## 2 Methods

### 2.1 Search strategy and data collection

In this study, CiteSpace (14) and VOSviewer (15) were employed for performance analysis and scientific mapping. The Web of Science Core Collection (WoSCC), as the most widely used multidisciplinary database in bibliometric analysis, was chosen as the data source. Following bibliometric analysis guidelines, the initial search terms were determined by reviewing the key terms used in relevant systematic reviews, after which the authors brainstormed additional terms (16). The final search strategy included two group of terms such as “Vagus Nerve Stimulation” and “Stroke,” combined using Boolean logic operators (see Appendix 1). The search and data collection were conducted in WoSCC on August 1st 2024, and limited to publications from January 1st 2004, to July 1st 2024. Only Original Articles and Review Articles published in English were included in the analysis.

### 2.2 Bibliometric analysis techniques

A bibliometric analysis of 191 eligible publications from 2000 to 2024 was conducted using CiteSpace and VOSviewer. VOSviewer was used to construct networks for analysing co-authorship by country, institution, and author. CiteSpace were applied to conduct reference co-cited analysis of reference and citation burst analysis from the keywords co-occurrence analysis. Microsoft Excel 2019 (Microsoft Corp., Redmond, WA, United States) was used to manage the data, create the figures of the annual number of publications and citations. To evaluate the impact factor of the publications included in this study, we gathered journal impact factors from the 2023 Journal Citation Report (JCR, Clarivate Analytics, Philadelphia, PA, United States).

## 3 Results

### 3.1 General performance and trends in scientific productivity

**Figure 1:**
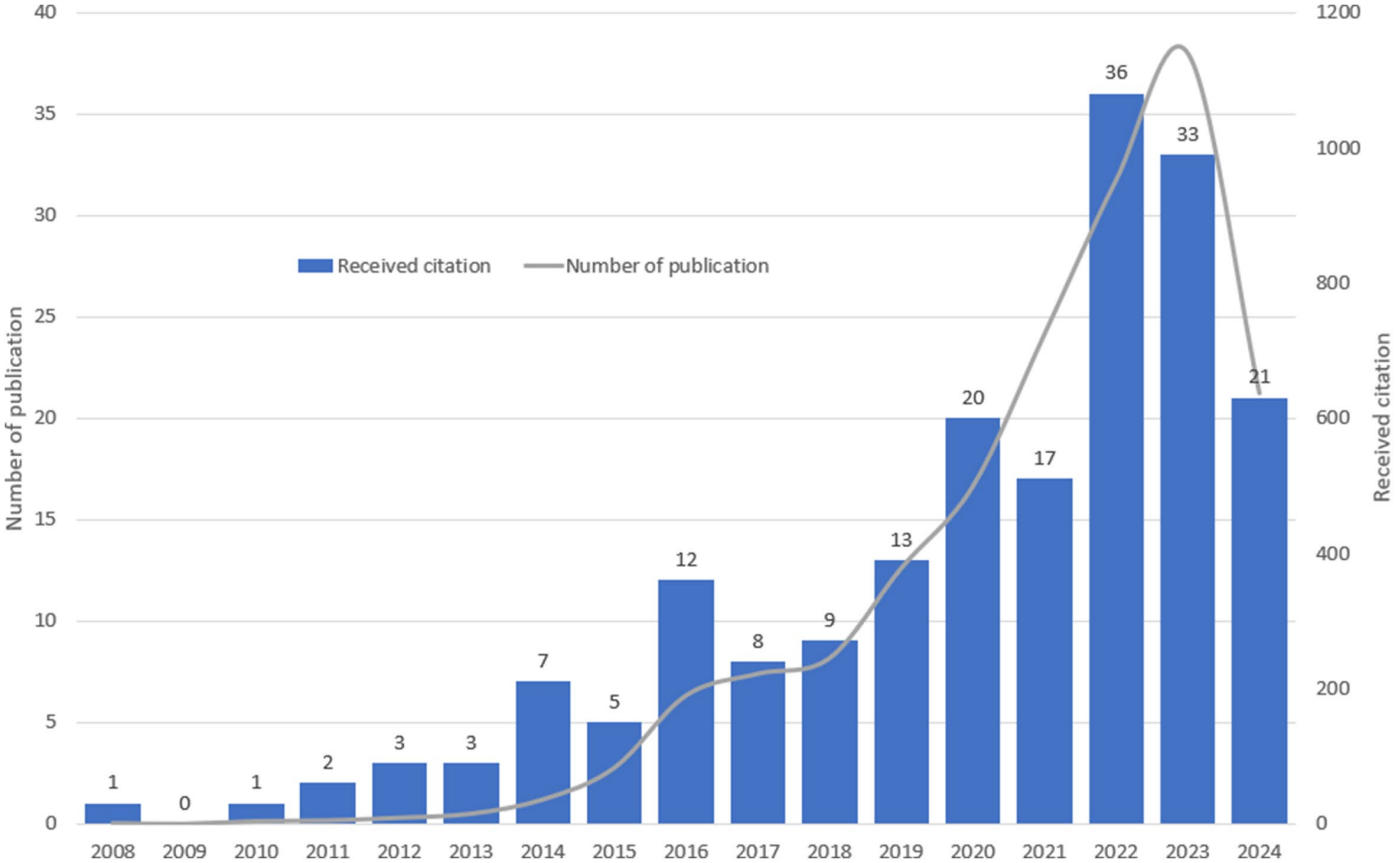
Annual number of publications and citations in the field of VNS for stroke (2008–2024).

A total of 305 documents were retrieved in this search. After excluding 27 non-English documents and 87 non-research types (e.g., Meeting Abstracts = 15, Editorials = 5, Letters = 4, Early Access = 3, Book Chapters = 2, Corrections = 1, News Items = 1, and Proceedings Papers = 1), 191 documents remained, consisting of 128 Original Articles and 63 Review Articles. Figure 1 illustrates the annual publication trends in the field of VNS for stroke from 2008 to 2024. The annual number of publications in this field has risen sharply over the past decade, increasing to over 10 in 2016 (12 publications) and peaking at 36 in 2022. The total citations for these publications amounted to 5,140, with an average of 26.9 citations per publication. The identified documents were indexed by 38 WoSCC categories, with the top five being: Neurosciences (95 publications), Clinical Neurology (70 publications), Rehabilitation (19 publications), Peripheral Vascular Disease (18 publications), and Medicine, Research & Experimental (12 publications). The top five agencies which providing the highest number of funding were the National Institutes of Health (NIH, 45 documents, United States), the U.S. Department of Health and Human Services (45 documents, United States), the National Natural Science Foundation of China (NSFC, 28 documents, China), the NIH National Institute of Neurological Disorders and Stroke (NINDS, 10 documents, United States), and MicroTransponder Inc. (9 documents, United States). Moreover, 142 of the 191 documents (74.3%) were open accessed.

### 3.2 Performance and the collaborative network of authors

**Figure 2:**
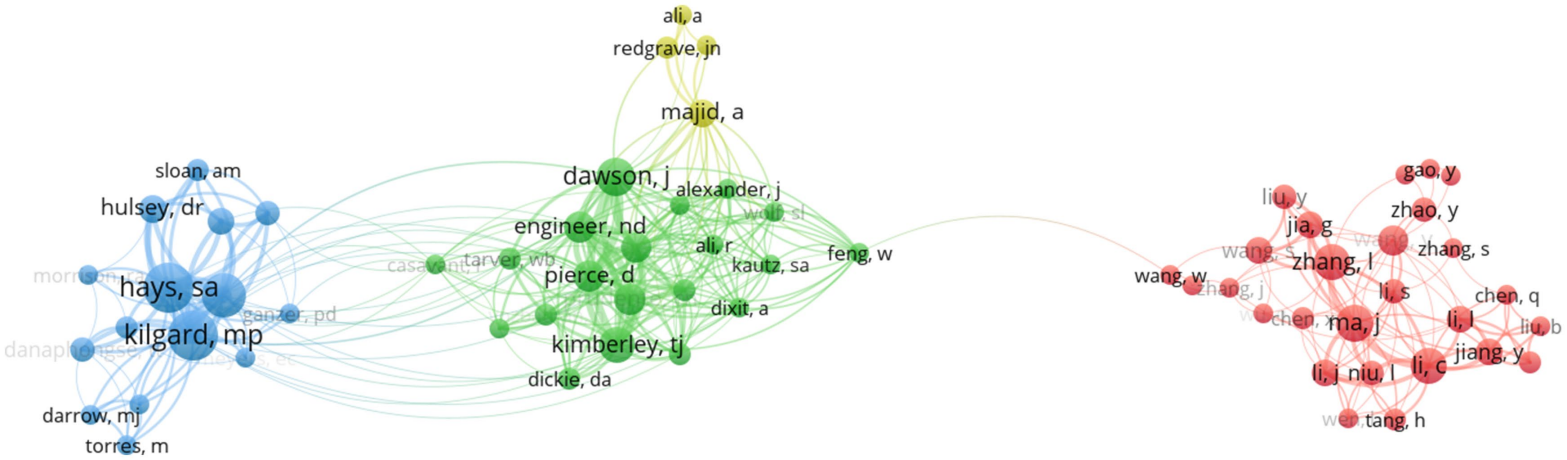
Collaborative network of authors in the field of VNS for stroke (2004–2024). (Produced by VOSviewer; each node represents an author, with 71 authors reaching the threshold of 3 publications; size of a node is determined by its total link strength; link between nodes is determined by the number of times the two authors appear in the same publication at the same time; larger nodes and thicker links represent more frequent collaboration between the two authors; nodes of the same colour represent a collaboration cluster).

A total of 885 authors contributed at least one publication in the field of VNS for stroke. Table 1 shows the top 11 authors with the highest number of publications, with Kilgard M. P., and Hays S. A. as the most prolific authors, each published 26 documents. They are followed by Rennaker R. L., with 20 publications. All three researchers are affiliated with The University of Texas at Dallas, USA. Among those most prolific authors, Rennaker R. L. achieved the highest average citation impact (ACI) of 70.1. Figure 2 presents the map of collaborative network of authors, with connections based on total link strength (TLS), representing how frequently two authors co-publish in one document. Four major collaborative networks were identified, with Ma J. (red cluster, 27 items) and Dawson J. (green cluster, 20 items) leading the two largest collaboration groups.

**Table 1:** Top 11 authors with the highest number of publications in the field of VNS for stroke (2008–2024).

Rank	Author	Quantity	ACI	TLS
=1	Kilgard M. P.	26	59	101
=1	Hays S. A.	26	55.9	95
3	Rennaker R. L.	20	70.1	89
4	Dawson J.	14	48	90
5	Ma J.	13	25.8	46
=6	Kimberley T. J.	12	51.2	85
=6	Li C.	12	38.1	49
=6	Zhang L.	12	25.7	37
=9	Pierce D.	9	58.6	79
=9	Engineer N. D.	9	47	77
=9	Prudente C. N.	9	47	77

ACI, average citation per item; TLS, total link strength (generated by VOSviewer).

### 3.3 Performance and the collaborative networks of countries/regions and institutions

**Figure 3:**
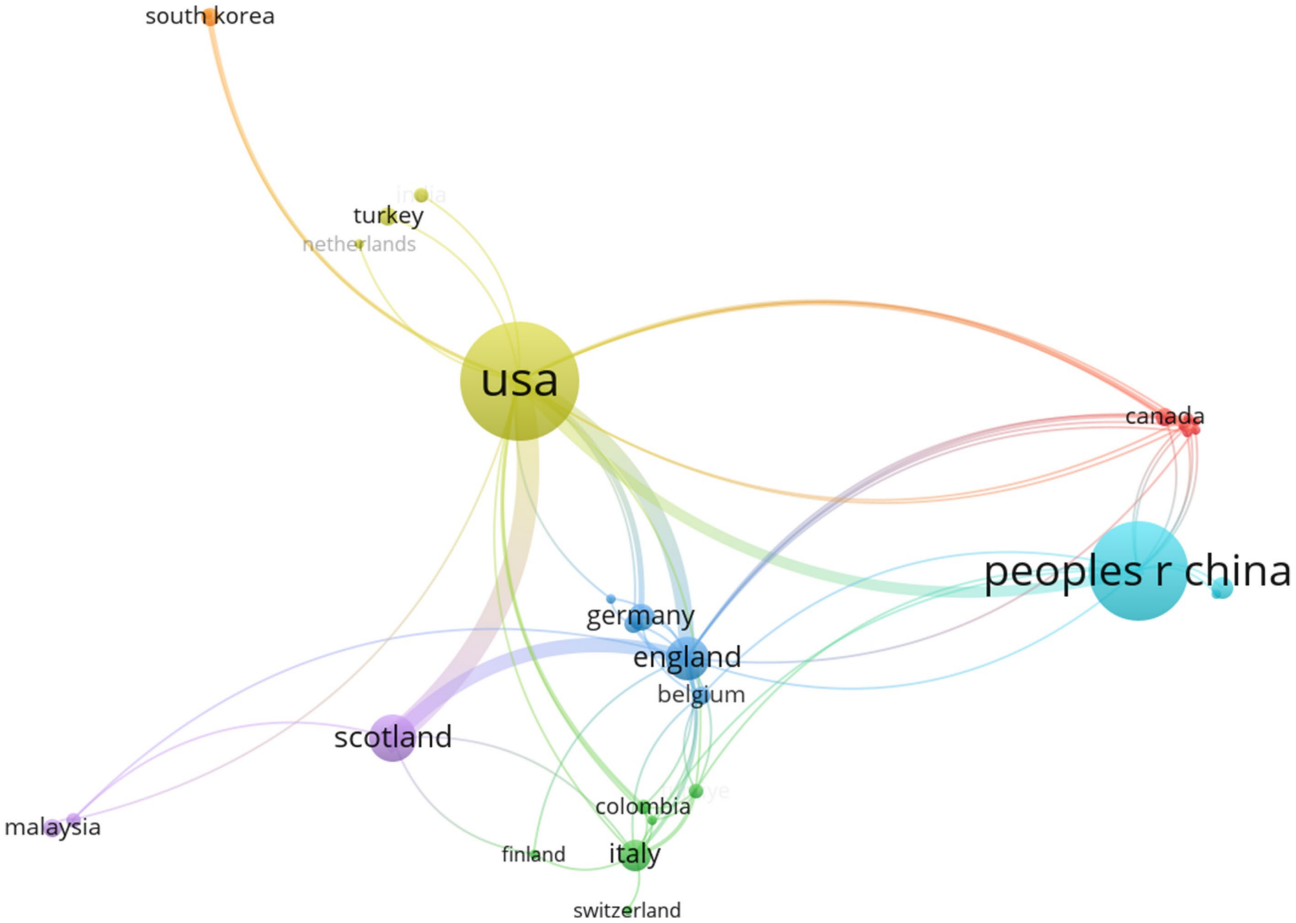
Collaborative network of countries/regions in the field of VNS for stroke (2008–2024). (Produced by VOSviewer; each node represents a country/region, with 33 countries/regions reaching the threshold of 1 publications; size of a node is determined by its total link strength; link between nodes is determined by the number of times the two countries/regions appear in the same publication at the same time; larger nodes and thicker links represent more frequent collaboration between the two countries/regions; nodes of the same colour represent a collaboration cluster).

**Figure 4:**
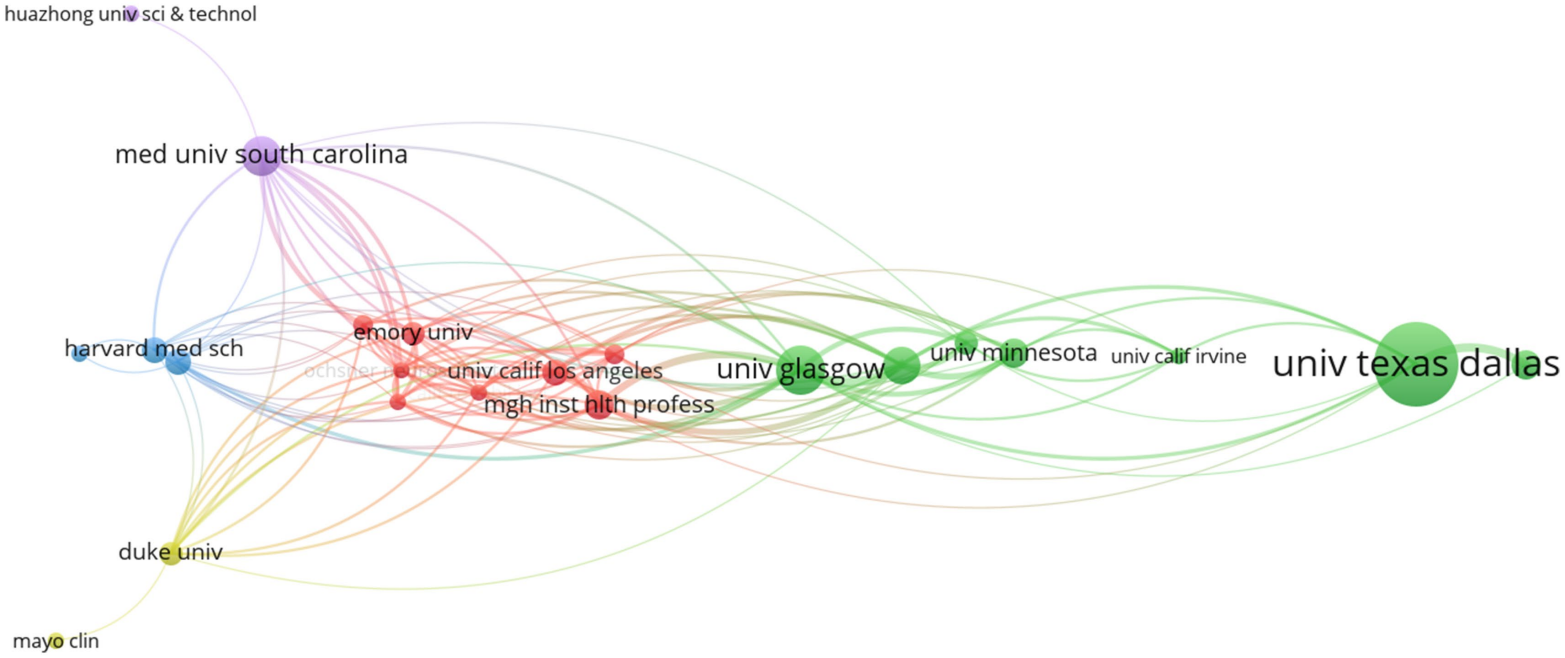
Collaborative network of institutions in the field of VNS for stroke (2004–2024). (Produced by VOSviewer; each node represents a institution, with 34 institutions reaching the threshold of 3 publications; size of a node is determined by its total link strength; link between nodes is determined by the number of times the two institutions appear in the same publication at the same time; larger nodes and thicker links represent more frequent collaboration between the two institutions; nodes of the same colour represent a collaboration cluster).

A total of 33 countries/regions within 356 institutions contributed to at least one publication in the field of VNS for stroke. Performance analyses by country/region and institution are shown in Tables 2, 3. The USA, China, and Scotland emerged as the most productive contributors, with 96, 69, and 17 publications, respectively. Among the 13 countries/regions with the highest number of publications, only China and Malaysia are developing countries. This is likely due to the high cost of VNS equipment, which is more widely used in developed nations. Scotland has the highest ACI, followed by England, indicating that their publications are cited most frequently in this field. The University of Texas at Dallas leads as the most productive institution, followed by Chongqing Medical University in China and the University of Glasgow in Scotland. The University of Minnesota received the highest ACI for papers on this topic. Figures 3, 4 presents the map of collaboration networks of countries/regions and institutions.

**Table 2:** Top 13 countries/regions with the highest number of publications in the field of VNS for stroke (2004–2024).

Rank	Country	Quantity	ACI	TLS
1	USA	96	35.4	52
2	China	69	17.3	18
3	Scotland	17	43.8	24
4	England	15	39.7	36
5	Italy	8	36.3	12
6	Germany	6	11	6
7	Japan	4	30.8	1
=8	Canada	3	11.3	9
=8	Belgium	3	25.3	6
=8	France	3	13.7	6
=8	South Korea	3	24.7	3
=8	Malaysia	3	1.7	1
=8	Turkey	3	11.3	1

**Table 3:** Top 11 institutions with the highest number of publications in the field of VNS for stroke (2004–2024).

Rank	Institution	Country	Quantity	ACI	TLS
1	University of Texas Dallas	USA	33	54.9	20
2	Chongqing Medical University	China	19	30.8	22
3	University of Glasgow	Scotland	15	37	54
=4	Medical University of South Carolina	USA	11	23.3	33
=4	University of Chinese Academic of Sciences	China	11	24.3	20
6	MicroTransponder Inc.	USA	10	44.9	45
7	Chongqing Key Lab Neurodegenra…	China	9	23.1	18
=8	MGH Institute of Health Professions	USA	7	40.4	36
=8	University of Minnesota	USA	7	81.1	24
=8	Texas Biomed Device CTR	USA	7	58.7	8
=8	Capital Medical University	China	7	15.6	3

In the map of collaboration networks of countries/regions, six collaboration clusters were identified, with USA and China led the two main clusters. Moreover, seven collaboration clusters of institutions were identified, with the University of Texas at Dallas and the University of Glasgow led the two primary clusters.

### 3.4 Performance of journals, categories, and funding agencies

Documents in this field has been published in 109 different journals. Table 4 highlights the 11 journals with the highest number of publications, with *Frontiers in Aging Neuroscience and Neurorehabilitation and Neural Repair* ranked first and second, respectively. *Brain Stimulation, Stroke, and Neural Regeneration Research* were journals with an impact factor over five in 2023. As can be seen from the type of journal, publications in the field of VNS for stroke are multidisciplinary and mainly focuses on the fields of neurology and rehabilitation medicine.

**Table 4:** Top 11 journals publishing the highest number of publications in the field of VNS for stroke (2004–2024).

Rank	Journal	Quantity	Country	2023 IF
1	Frontiers in Neuroscience	13	Switzerland	3.2
2	Neurorehabilitation and Neural Repair	10	USA	3.7
=3	Brain Stimulation	9	USA	7.6
=3	Frontiers in Neurology	9	Switzerland	2.7
5	Stroke	7	USA	7.8
7	Autonomic Neuroscience Basic Clinical	5	Holland	3.2
=7	Journal of Stroke Cerebrovascular Diseases	4	USA	2
=7	Neural Regeneration Research	4	China	5.9
=9	Behavioural Brain Research	3	Holland	2.6
=9	Experimental Neurology	3	USA	4.6
=9	Medical Science Monitor	3	USA	2.2
=9	Neural Plasticity	3	USA	3
=9	Scientific Reports	3	England	3.8

ACI, average citation per item represents the average citations of that journal’s publications; 2023 IF, 2023 Journal Impact Factor from Journal Citation Reports^™^.

### 3.5 Analysis of reference

The top 10 RCTs which received the highest number of citations in the field of VNS for stroke are shown in Table 5. Among them, Dawson J. et al. (2021) entitled: “Vagus nerve stimulation paired with rehabilitation for upper limb motor function after ischaemic stroke (VNS-REHAB): a randomized, blinded, pivotal, device trial” received the highest number of citations. All these RCTs investigate motor function rehabilitation after stroke, with most focusing on upper limb function, and only one study exploring post-stroke sensation and emotion.

**Table 5:** Top 10 RCTs with the highest number of citations in the field of VNS for stroke (2008–2024).

Study	Citation	Country	Participants (total number, treat group age, *n*% male), placebo group (age, *n*% male)	Type of stroke	Stroke location	Time of onset of stroke	Method of electrode implantation	Site of electrode implantation intervention (intensity, pulse, pulse duration, sessions)	Control	Outcome measures	Follow-up
Dawson et al. (17)	250	USA and UK	108, 53 (59.1 ± 10.2, 64% male), 55 (61.1 ± 9.2, 65.5% male)	Ischaemic stroke	Unilateral supratentorial	9 months to 10 years	Invasive VNS	0.8 mA, 0.1 ms, 30 Hz stimulation pulses, lasting 0.5 s, 3 sessions per week, 6-week	Sham stimulation + rehabilitation therapy + home exercise during follow-up period	FMA-UE, WMFT, MAL, SIS, SS-QOL, EQ-5D, BDI	90 days
Dawson et al. (18)	220	UK	20, 9 (57.9 ± 17.2, 77.8% male), 11 (60.7 ± 10.7, 81.8% male)	Ischemic stroke	Unilateral supratentorial	≥6 months	Invasive VNS	0.8 mA, 0.1 ms, 30 Hz stimulation pulses, 2-h sessions, 3 sessions per week, 6-week	Rehabilitation alone	FMA-UE, ARAT, grip, and pinch strength, SIS, Box and Block Test, and 9-hole peg test	30 days
Kimberley et al. (19)	126	USA	17, 8 (59.5 ± 7.4, 50% male), 9 (60.0 ± 13.5, 56% male)	Ischemic stroke	Unilateral supratentorial	4 months to 5 years	Invasive VNS	0.8 mA, 0.1 ms, 30 Hz stimulation pulses, 2-h sessions, 3 sessions per week, 6-week	Sham stimulation + rehabilitation therapy + home exercise during follow-up period	FMA-UE, WMFT, MAL, Box and Block Test, 9-Hole Peg Test	90 days
Capone et al. (20)	93	Italy	12, 7 (53.71 ± 5.88, 57% male), 5 (55.60 ± 7.12, 60% male)	Ischemic or haemorrhagic stroke	Unspecified	≥1 year	Non-invasive VNS	Mean 2–4.5 mA, lasting 30 s and composed by 600 pulses intratrain pulse frequency = 20 Hz; pulse duration = 0.3 ms repeated every 5 min for 60 min, for 10 days	Sham stimulation + rehabilitation therapy	Fugl-Meyer, NIHSS, BI, Modified Rankin, Modified Ashworth Scale	1 day
Wu et al. (21)	67	China	21, 10 (64.50 ± 9.97, 50% male), 11 (61.82 ± 10.63, 73% male)	Ischemia stroke	Unspecified	0.5 month to 3 months	Non-invasive VNS	20 Hz; 0.3 ms pulse width, lasting 30 s, once every 5 min, 30 min per day for 15 consecutive days	Sham stimulation + rehabilitation therapy	FMA-U, WMFT, FIM, Brunnstrom	12 weeks
Dawson et al. (22)	46	USA and UK	18, 8 (59.5 ± 7.3, 50% male), 9 (63 ± 12.0, 44% male)	Ischemic stroke	Unilateral supratentorial	4 months to 5 years	Invasive VNS	0.8 mA, 0.1 ms, 30 Hz stimulation pulses, 30 min sessions, 3 sessions per week, 6 weeks	Sham stimulation + rehabilitation therapy+ home exercise for 90 days	FMA-UE, WMFT; Box and Block Test, Nine-Hole Peg Test, SIS, MAL	1 year
Li et al. (23)	39	China	60, 30 (69.2 ± 12.3, 50% male), 30 (68.3 ± 12.1, 47% male)	Ischemic or haemorrhagic stroke	Unspecified	Acute stroke patients	Non-invasive VNS	Mean 1.71 ± 0.5 mA, 0.3-ms square pulses at 20 Hz for 30 s, repeated every 5 min, 20 min a day for 20 working days (5 days a week for 4 weeks)	Sham stimulation + rehabilitation therapy	WMFT, FMA-U, FMA-L, FMA-S, Fugl-Meyer, SIS, HADS	1 year
Chang et al. (24)	38	USA	36, 18/18 (mean 59.02 ± 1.98, gender unspecified)	Stroke	First single focal unilateral supratentorial stroke	≥6 months	Non-invasive VNS	0.1 to 5.0 mA, single 500 ms bursts, 30 Hz, pulse width = 0.3 ms, 1 h sessions, 3×/week for 3 weeks	Sham stimulation + Robotic training	Instrumental Surface Electromyography Assessment, UE-FM, MRC, WMFT, MTS	1 day
Badran et al. (9)	19	USA	16, 9 (57.33 ± 8.28, 44% male), 7 (58.71 ± 6.45, 71% male)	Ischemic or haemorrhagic stroke	Unspecified	≥6 months	Non-invasive VNS	3–25 mA, 25 Hz, lasting 5 s, 1 to 1.5 h, 3 sessions per week for 4 weeks	Sham stimulation + rehabilitation therapy	FMA-UE, WMFT	8 weeks
Dawson et al. (25)	18	USA and UK	108, 53 (59.1 ± 10.2, 64% male), 55 (61.1 ± 9.2, 65.5% male)	Ischemic stroke	Unspecified	≥9 months	Invasive VNS	0.8 mA, 100 μs, 30 Hz stimulation pulses, lasting 0.5 s, 3 sessions per week for 6 weeks	Sham stimulation + rehabilitation therapy	FMA-UE WMFT	90 days

**Figure 5:**
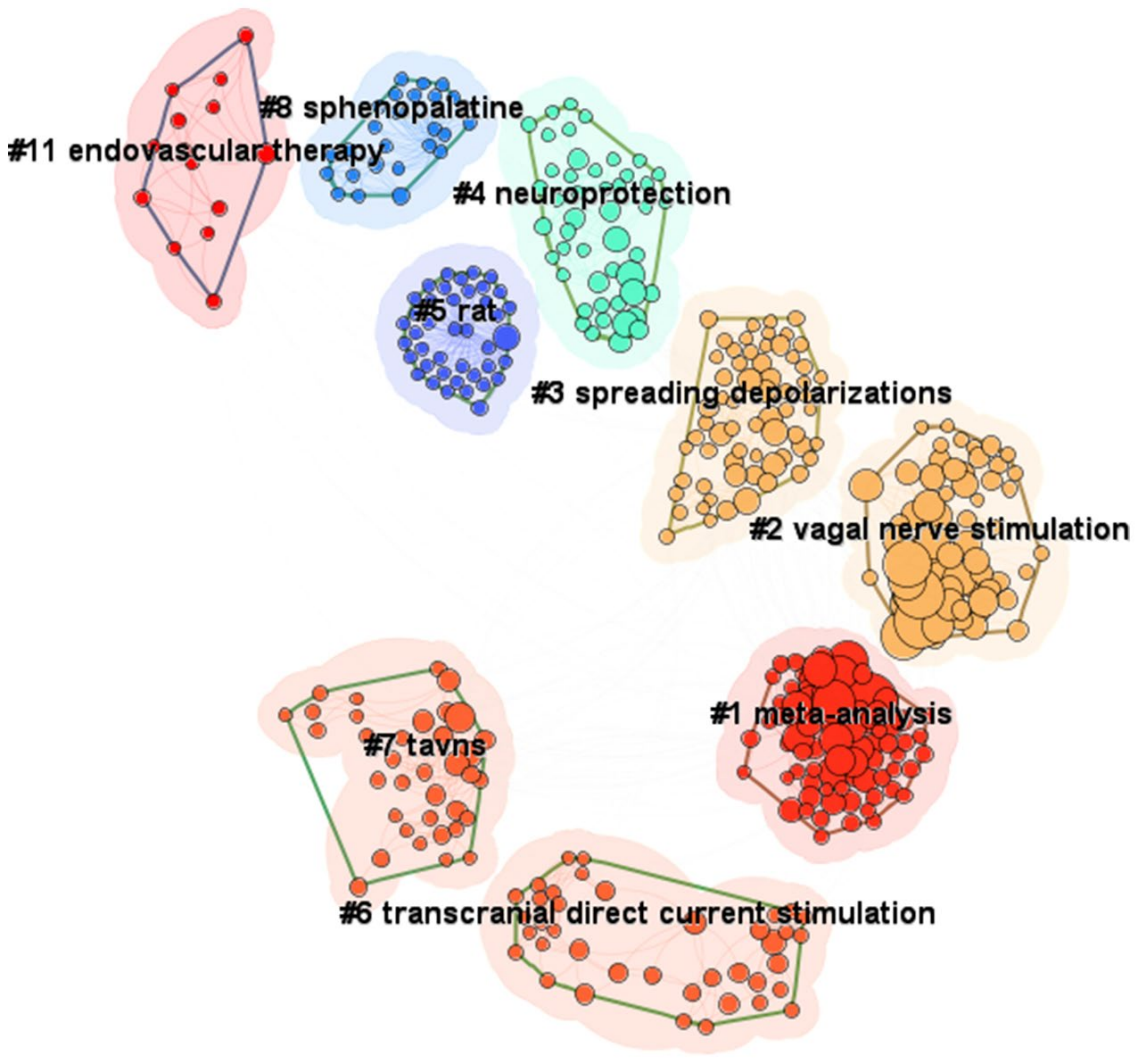
Knowledge structure of the field of VNS for stroke, based on the reference co-citation analysis (2004–2024). (Produced by CiteSpace; time slicing = 01/01/2004 to 01/07/2024, year per slice = 1; from left to right represents the evolution of the research clusters from 2004 to 2024; each cluster is made up of its contained publications represented by nodes, and the size of the cluster is determined by the number of publications it contains).

CiteSpace was used to visualize both the reference co-cited analysis, and the citation bursts analysis from the keywords co-occurrence analysis in the field of VNS for stroke. A total of 11 clusters were identified in Figure 5. The research trends over the past 20 years are illustrated in the Figure 5, spanning from 2008 on the left to 2024 on the right. Two decades ago, VNS was predominantly an invasive procedure, involving the surgical placement of electrodes directly on the vagus nerve. Early studies focused on the mechanisms of VNS, particularly in cluster #11 endovascular therapy (26), followed by basic experimental research in cluster #5 rat models. Around the same time, transauricular VNS (cluster #7 taVNS) emerged and gradually found clinical application. Later, non-invasive brain stimulation methods, such as #6 transcranial direct current stimulation (tDCS), which is similar to VNS, were introduced for stroke treatment. Research has shown that VNS exerts cluster #4 neuroprotective effects by activating anti-inflammatory pathways (27). Further studies discovered that cluster #3 spreading depolarization (SD) occurs during acute cerebral infarction, and VNS can reduce cluster #3 SD frequency following focal ischemia (28), leading to stroke recovery. In recent years, researchers have conducted cluster #1 meta-analyses of previous evidence, consolidating the body of knowledge on VNS for stroke.

To determine the robustness of the knowledge structure and the turning point paper derived from the reference co-citation analysis, a sensitivity analysis was performed. Appendix 2 shows the results of the reference co-citation analysis with an adjusted time slice from 1 year to 5 years, which demonstrates the robustness of the previous findings.

### 3.6 Analysis of keywords

**Figure 6:**
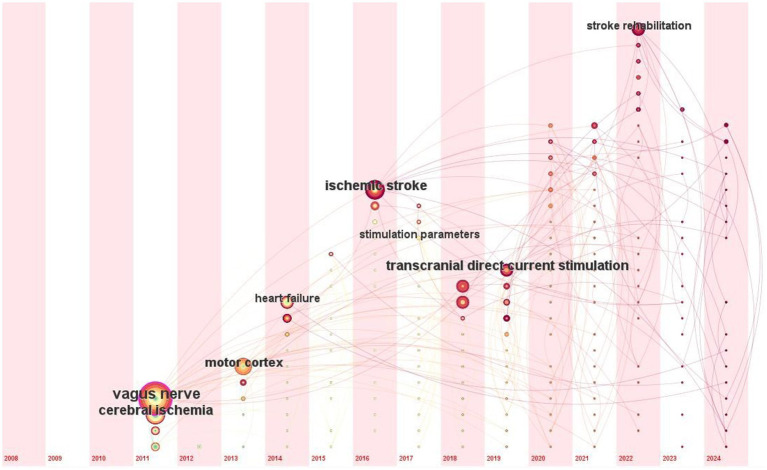
Time zone view of keyword co-occurrence analysis in the field of VNS for stroke (2008–2024). (Produced by CiteSpace; time slicing = 01/01/2004 to 01/07/2024, year per slice = 1; from left to right represents the evolution of the authors’ keywords from 2008 to 2024).

Analysing the most frequently mentioned keywords helps to identify emerging research trends. Figure 6 shows time zone view of keyword co-occurrence analysis in the field of VNS for stroke. Since 2011, VNS has evolved from focusing on *cerebral ischemia* to gradually shifting toward *stroke rehabilitation*. Researchers have explored the effects of VNS on the *motor cortex*, and its application in the treatment of *heart failure* has also been gradually investigated. In recent years, VNS research has increasingly concentrated on rehabilitation for *ischemic stroke*, with continuous optimization of *stimulation parameters*. At the same time, the development of non-invasive neuromodulation methods, such as *transcranial direct current stimulation (tDCS)*, has opened up new avenues for VNS research.

**Figure 7:**
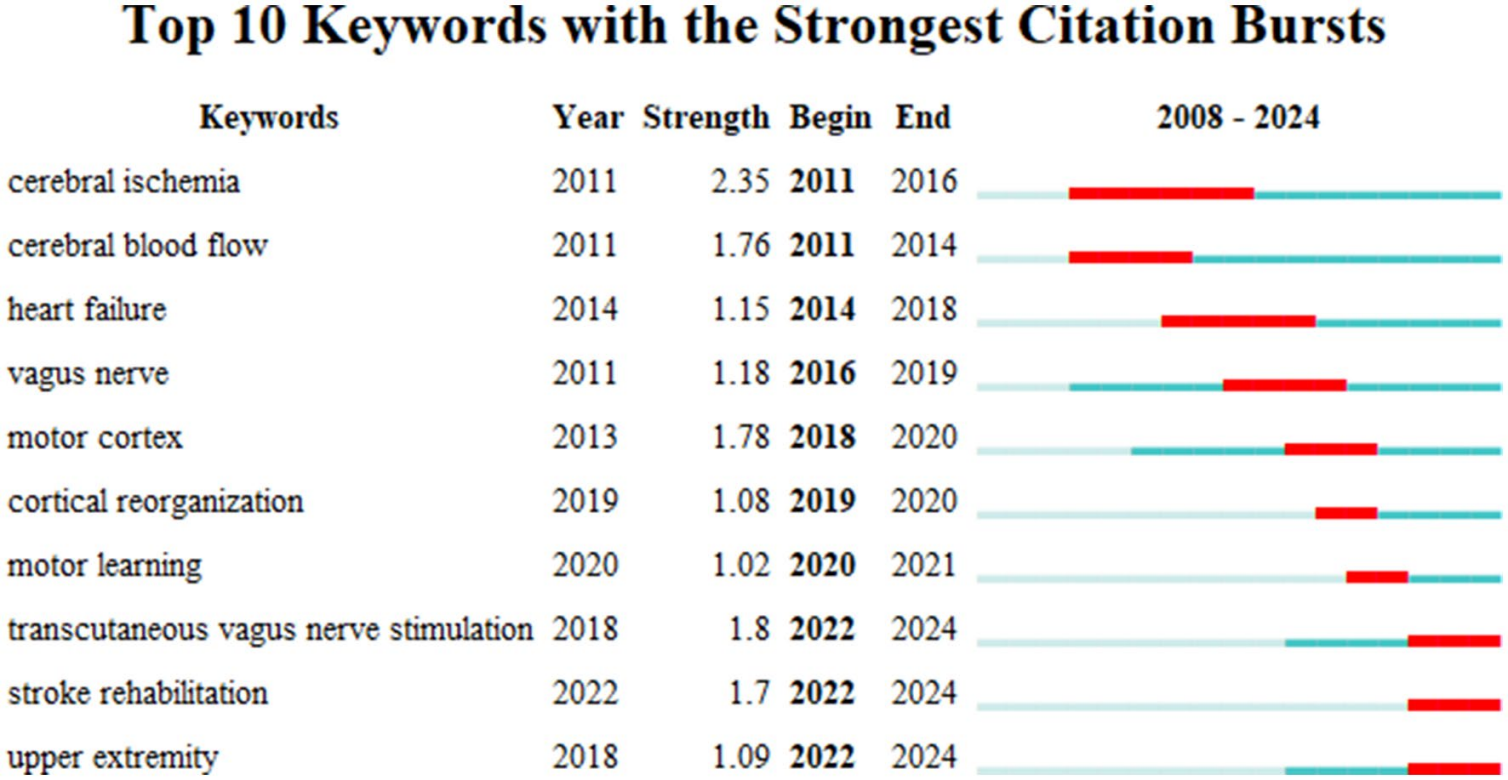
Top10 keywords with the strongest citation bursts in the field of VNS for stroke (2008–2024).

Figure 7 displays the top 10 author’s keywords with the strongest citation bursts, as revealed by the keyword co-occurrence analysis. Over the past 20 years, terms like cerebral ischemia, cerebral blood flow, heart failure, vagus nerve, and motor cortex have exhibited the most significant citation bursts, indicating that these topics have garnered considerable attention from researchers in this field. In the past 5 years, keywords such as cortical reorganization, motor learning, transcutaneous vagus nerve stimulation, stroke rehabilitation, and upper extremity have emerged, suggesting that these areas may be at the forefront of current research. This suggests that research is gradually shifting from investigating pathophysiological mechanisms to exploring functional recovery and remodelling. There is also a clear trend from basic research toward translational studies, with an increasing focus on neuroplasticity and neuromodulation technologies.

## 4 Discussion

In this study, we visualize and analyse 191 documents authored by 885 researchers from 365 institutions across 33 countries. These documents were published in 109 journals and indexed in 38 categories within the WoSCC. This bibliometric analysis examines the evolution of the knowledge structure and emerging research trends in VNS for stroke from 2004 to 2024.

### 4.1 Analysis of the current status of research in the field of VNS for stroke

The results indicate that annual number of publications in the field of VNS for stroke has steadily increased since the 2008, peaking in 2022–2023. This trend suggests that the field has significant development potential in the future. By analysing the number of authors’ publications and their collaboration networks, it was found that the three most published authors, Kilgard M. P., Hays S. A., and Rennaker R. L., and their teams developed a technology for targeted plasticity therapy, now known as pair VNS (29). Pair VNS with rehabilitation training to improve the plasticity of the corticospinal motor network, thereby improving synaptic connections to muscles involved in rehabilitation. In the rat model, it was found that pairing VNS with movement or auditory stimuli could enhance plasticity in the motor and auditory cortices, respectively (30). In a mouse model, auditory training combined with VNS-induced plastic reorganization of the auditory cortex, leading to an improvement in tinnitus symptoms (31). Furthermore, VNS combined with exercise training significantly improves motor function in patients compared to exercise training alone. Research indicates that moderate intensity VNS can boost motor cortex plasticity, while high intensity VNS may have adverse effects (32) Beyond facilitating motor function recovery, VNS has been shown to promote the reduction of olfactory and auditory fear responses (33) and improve anxiety levels (34). When combined with tactile rehabilitation, VNS significantly enhances recovery of sensory and motor functions after peripheral nerve injuries (35). Furthermore, the team explored optimizing VNS parameters and the duration of stimulation based on individual neural activity to achieve better outcomes (36).

Dawson et al. (17) conducted several clinical trials, including pivotal, randomized, triple-blind, multi-site, sham-controlled studies (19, 37). These studies demonstrate that VNS combined with rehabilitation significantly improves moderate to severe upper extremity weakness resulting from chronic ischemic stroke and exhibits good long-term compliance (38). The VNS aids in the brain’s relearning and restoration of damaged motor functions by enhancing nervous system plasticity.

### 4.2 Research trends in the field of VNS for stroke

Through performance analysis and science mapping, the current research trends were summarised.

First, studies indicate that the biological mechanism of VNS in the treatment of stroke primarily involves the inhibition of inflammatory pathways activated after stroke, thus reducing secondary damage. Specifically, VNS has been shown to effectively decrease the release of inflammatory cytokines (39), stabilise endothelial cell function (40), maintain the integrity of the blood-brain barrier (41), and mitigate excitotoxicity resulting from stroke (42). By increasing the release of neurotransmitters such as norepinephrine and acetylcholine, VNS promotes prominent plasticity and long-term enhancement, contributing to the reorganization and recovery of motor and cognitive functions (43). In the clinical setting, VNS shows potential for promoting neuroplasticity and functional recovery, particularly in the rehabilitation of chronic stroke patients. Numerous studies have shown that when VNS is combined with rehabilitation training, it significantly improves the plasticity of neural networks and facilitates the reconstruction of damaged neural pathways, thus accelerating the recovery of motor functions (30, 31, 38). Despite the valuable information provided by existing studies on the role of VNS in stroke treatment, the underlying neurophysiological mechanisms of VNS remain not fully elucidated. In particular, the specific mechanisms of action in different types of strokes and at various recovery stages require further exploration through more in-depth experimental and clinical studies.

Secondly, traditional VNS requires surgical implantation of a device. Although VNS received approval from the US FDA for clinical treatment as early as the last century, this invasive method is associated with certain adverse effects, including paralysis of the vocal cord and dysphagia (44, 45). Moreover, patients often face high medical costs (46). In recent years, the emergence of non-invasive VNS methods, such as taVNS and tcVNS, has provided patients with a safer and more cost-effective alternative approach (47). According to Peuker’s anatomical study of 22 cadavers, the auricular branch of the vagus nerve (ABVN) was 100% distributed in the cymba of the auricle. Furthermore, there was a notable distribution of ABVN in other regions of the ear, including the crus of the helix, the tragus, and the antihelix (48). This discovery offers an anatomical basis for the development of non-invasive VNS. In traditional Chinese medicine, ear acupuncture theory and ear acupuncture emphasise stimulating specific areas of the ear to positively influence bodily health. ABVN covers the primary bundle of the vagus nerve and stimulates the human ear, allowing the stimulation to travel through the vagal bundle and reach the brain via the brainstem. This results in a notable increase in activation in areas such as the angular gyrus, caudate, cerebellum, cingulate, and frontal cortex (49). Stimulating these regions may induce changes in the activity of vagus nerve pathways, affecting the structures of the brainstem and central nervous system, and producing regulatory effects similar to those of the invasive VNS without the need for surgery (50). The reported side effects are mainly related to the administration of transcutaneous electrical current, causing local skin irritation. In general, tVNS is safe and well tolerated at the doses tested in research studies (51). This non-surgical treatment approach is gaining traction, particularly with taVNS. Future research will focus on optimizing the design of non-invasive VNS devices, exploring their effectiveness in stroke rehabilitation, and comparing them with traditional implantable VNS (51). The advancement of this technology offers patients new rehabilitation options and is expected to reduce treatment risks and costs.

Thirdly, the combination of VNS with other rehabilitation techniques is emerging as a significant research direction, particularly regarding the synergistic effects in multimodal treatment. Studies have demonstrated that VNS combined with rehabilitation training significantly enhances the recovery of upper limb motor function in patients with stroke. For instance, an one-year observational study (22) and a multicenter, three-year observational study (52) found that VNS combined with rehabilitation was more effective than with traditional rehabilitation methods (38). Furthermore, integration of upper limb robotic training with selectively applied auricular VNS significantly reduced wrist and hand spasticity during upper limb extension training in stroke patients with stroke (24). This combined approach has also been shown to improve arm function in individuals with chronic stroke (20). In another study, Szulczewski (53) reported that combining taVNS with controlled slow breathing further enhanced therapeutic effects. Future research will focus on exploring the synergy between VNS and different exercise training modalities, particularly optimizing stimulation parameters to maximise functional recovery and neuroplasticity while minimising potential side effects (36, 54). This exploration of precision treatment will offer new avenues for the combined application of VNS and various rehabilitation technologies, with the potential to deliver more effective rehabilitation programmes for stroke patients. These findings highlight the complementary nature of VNS with other rehabilitation technologies in addressing various stroke symptoms. Specifically, when VNS is combined with neurorehabilitation robots, it may produce even greater therapeutic benefits. The synergistic effects of transcranial direct current stimulation (tDCS) and transcutaneous vagal nerve stimulation (taVNS) on brain response are also being investigated (55). Although it is rarely used in stroke, VNS combined with other neuromodulation techniques (e.g., BCI, TMS, VR based rehabilitation) would provide insight into future innovations.

Furthermore, while there is a substantial body of research on the recovery of motor and sensory functions after stroke using VNS, studies addressing other functional disorders are relatively scarce, such as cognitive impairment, speech disorders, and swallowing difficulties. These areas are poised to become important research directions in the future. Post-stroke cognitive impairment is often the result of hypoperfusion, ischemia, hypoxia, and neuroinflammation associated with chronic stroke. The VNS has the potential to improve cognitive impairment by initiating anti-inflammatory mechanisms (56). However, most existing studies on the cognitive effects of VNS focus on healthy individuals, and its efficacy in stroke patients still requires validation through large-scale clinical trials to confirm both effectiveness and safety. In the study of swallowing disorders following stroke, Long L’s team discovered that transauricular VNS (taVNS) can slightly improve dysphagia in rats after ischemic stroke. This improvement is achieved primarily by promoting myelin regeneration, inducing angiogenesis, and inhibiting inflammatory responses in rat white matter (57). Similarly, Wang et al. (58) demonstrated that transcutaneous ear VNS can help improve dysphagia after acute stroke. Despite these findings, there remains a lack of studies on the application of VNS in post-stroke dysphagia, which requires further exploration and validation of the existing clinical evidence. Regarding aphasia resulting from stroke, VNS may facilitate the recovery of language functions by modulating brain areas associated with language (59). A study by Morrison et al. (60) indicated that when VNS is paired with mandibular movement, it can enhance the plasticity of the oral-facial neural network, potentially serving as an auxiliary method to rehabilitate motor aphasia after stroke. However, the efficacy of VNS on sensory aphasia and mixed aphasia remains unclear, highlighting a gap in relevant research that warrants further investigation. Since each stroke patient experiences varying degrees of neurological damage and functional rehabilitation needs, exploring the efficacy of VNS across different types of functional disorders will be a central focus of future research. By further investigating the role of VNS in cognitive, language, swallowing, and other functional disorders, researchers hope to develop more precise treatment strategies to improve rehabilitation outcomes across various dimensions after stroke.

Finally, despite being an important brain stimulation technology, in addition to treating stroke, has been used as a potential therapy for epilepsy, depression, anxiety, chronic heart failure, and fibromyalgia (44), there is currently no consensus on the “optimal” stimulation parameters for VNS. The interplay of various stimulation parameters, such as current intensity, pulse width, frequency, duty cycle, and duration, complicates the clinical application of VNS (61). However, as research into VNS theory and application deepens, future studies may leverage neuroimaging methods such as functional magnetic resonance imaging (fMRI) and positron emission tomography (PET), to monitor the brain’s response to VNS. These technologies allow for real-time observation of changes in brain activity and neural networks, providing valuable insights into the effects of VNS on different brain regions (62). Furthermore, the application of biomarkers can support personalised treatment approaches. By analysing patients’ gene expression, inflammation levels, and other biological indicators, researchers can better understanding of the mechanisms of VNS in various patients. To be specific, gene expression can help researchers identify genes and pathways that influence neuroregulation, and inflammation levels can help assess how well VNS regulates the immune system. A meta-analysis showed that taVNS was effective in modulating IL-1ß and IL-10, iVNS modulated IL-6 (63). Other biological indicators, such as hormone levels and neurotransmitters, can provide insight into the functional state of the nervous system. By integrating these data, researchers can determine each patient’s unique response to VNS treatment, thereby identifying the most suitable parameter settings and rehabilitation strategies for individualised treatment (64). By integrating neuroimaging and biomarkers, future research is expected to develop more precise stimulation parameters and personalised rehabilitation programmes. This approach not only aims to optimise the efficacy of VNS but may also can improve overall functional recovery in stroke patients, encompassing motor, cognitive, and other areas. A deeper exploration of this direction will pave the way for personalised application of VNS, making it more targeted and effective in clinical practice.

### 4.3 Limitations

This bibliometric analysis identifies potential collaborators, institutions, research hotspots, and future trends in the field of VNS for stroke. Our findings will serve as a valuable guide for clinicians, researchers, industry partners, policymakers, and other stakeholders. However, this study has some limitations.

First, our data were extracted solely from WoSCC, which may have overlooked relevant studies published in other databases, such as PubMed, Scopus, and Embase. Nonetheless, WoSCC is a comprehensive and multidisciplinary database, making the results of this study highly reliable. Second, data collection was restricted to papers published on August 1, 2024. Given that VNS is currently a hot research topic with a wealth of related literature and considering that the WoSCC database is continuously updated, papers published after August 1, 2024, were not included in this analysis. This limitation may affect our comprehensive understanding of the latest research trends in the field.

## 5 Conclusion

To our knowledge, this is the first bibliometric analysis focused on VNS for stroke. The annual number of publications in this field has steadily increased over the past 20 years. Future research hotspots and trends primarily include the following aspects: Investigating the neurophysiological mechanisms underlying vagus nerve therapy for stroke. Exploring the synergistic effects of VNS combined with various rehabilitation training modalities. Conducting comparative studies between non-invasive VNS and invasive VNS. Assessing the efficacy and safety of VNS for other functional disorders beyond post-stroke motor rehabilitation. Determining the “optimal” parameters for VNS in stroke rehabilitation. Current research shows regional imbalances, and it is suggested that closer collaboration between countries, researchers, and institutions will enhance the development of personalised VNS treatment and rehabilitation programmes for stroke patients.

## Data Availability

The original contributions presented in the study are included in the article/Supplementary material, further inquiries can be directed to the corresponding author.
