# Supplementary material for: Two decades of vagus nerve stimulation for stroke: a bibliometric analysis
Source: Front Neurol. 2025 Apr 4;16:1531127. doi: 10.3389/fneur.2025.1531127 (PMC12006007; doi:10.3389/fneur.2025.1531127)
Supplement: Supplementary file 1 [file Table_1.docx]

**Appendix 1.** Supporting information

#1 Vagus Nerve Stimulation

TI/AK=(“Vagus Nerve Stimulation” OR “Vagus Nerve Stimulation” OR “VNS”)

#2 Stroke

TI/AK=(Stroke OR “Brain Infarction” OR “Brain Stem Infarctions” OR “Cerebral Infarction”)

#1 AND #2

Timespan: 2004-01-01 to 2024-07-01

**Appendix 2.**


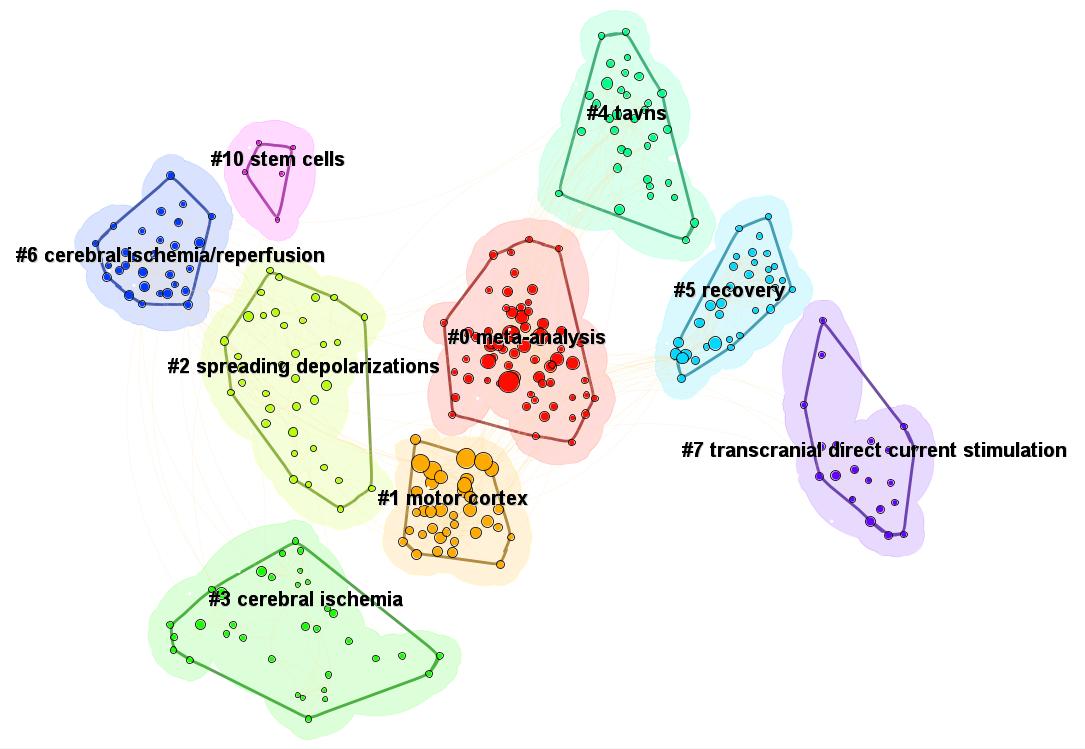


Knowledge structure of the field of VNS for stroke, based on the reference co-citation analysis (2008 - 2024)

(Produced by CiteSpace; Time slicing=01/01/2004 to 01/07/2024, year per slice=5; From left to right represents the evolution of the research clusters from 2008 to 2024; each cluster is made up of its contained publications represented by nodes, and the size of the cluster is determined by the number of publications it contains)
